# Interplay of periglomerular and granule cell inhibitory synapses on mitral cell spiking

**DOI:** 10.1186/1471-2202-12-S1-P269

**Published:** 2011-07-18

**Authors:** Denise Arruda, Rodrigo Publio, Antonio C Roque

**Affiliations:** 1 Departmento de Física, FFCLRP, Universidade de São Paulo, Ribeirão Preto, SP, 14040-901, Brazil

## 

**Figure 1:**
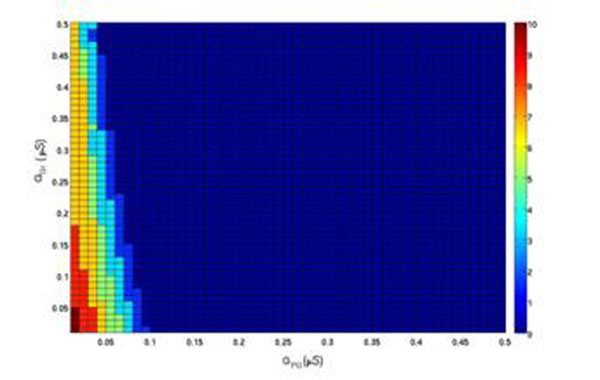
Firing rate of mitral cell as a function of maximal conductances of inhibitory synapses made by PG (horizontal axis) and Gr (vertical axis) cells. The color-code bar on the right indicates frequency in Hz.

Mitral cells in the olfactory bulb (OB) receive dendrodendritic inhibitory synaptic inputs at two different levels of OB: at the glomerular level, inhibitory input is provided by periglomerular (PG) cells and, at a deeper level (the external plexiform layer), inhibitory input is provided by granule (Gr) cells [1]. It is thought that both kinds of inhibition control mitral cell spiking in specific ways: PG early inhibition would regulate spike initiation while Gr inhibition would coordinate spike synchronization among mitral cells [1,2]. However, recent computational modeling studies have suggested that, although acting at separate locations, PG and Gr inhibitory synapses may have integrated effects on mitral cell spiking which may be beneficial for odor representation and processing [2]. In this work we constructed in NEURON [3] a model of a triad composed of reduced compartmental models of a mitral cell, a PG cell and a Gr cell to study the combined effect of the inhibitory dendrodendritic synapses of the PG and Gr cells on the firing frequency of the mitral cell. The mitral and Gr cell models were taken from [4] with all parameters and characteristics kept as in the original. The PG cell model was based on the five-compartment model used in [5]. The cells were coupled by models of their reciprocal dendrodendritic synapses. The main parameters investigated in our simulations were the conductances of the dendrodendritic synapses, the amplitudes, durations and initiation times of the excitatory inputs applied to the mitral and PG cells representing stimuli coming from olfactory sensory neurons, and the amplitude, duration and initiation time of the input applied to the Gr cell representing afferents from other cells in the olfactory bulb. We performed simulations in which these parameters were varied and studied their effects on the mitral cell’s spiking pattern. Our results show that the response pattern of the mitral cell is strongly dependent of the two inhibitory synapses, and that their interaction can regulate both the spike frequency and timing of the mitral cell. Figure 1 illustrates one of these results.
